# Prognostic significance of estrogen, progesterone and HER2 receptors’ status conversion following neoadjuvant chemotherapy in patients with locally advanced breast cancer: Results from a tertiary Cancer Center in Saudi Arabia

**DOI:** 10.1371/journal.pone.0247802

**Published:** 2021-03-05

**Authors:** Khalid Al-Saleh, Tareq Salah, Maria Arafah, Sufia Husain, Ammar Al-Rikabi, Nashwa Abd El-Aziz

**Affiliations:** 1 Division of Hematology-Oncology, Department of Medicine, College of Medicine, King Saud University, Riyadh, Saudi Arabia; 2 Division of Radiation Oncology, Oncology Center, King Saud University, Riyadh, Saudi Arabia; 3 Department of Clinical Oncology and Nuclear Medicine, Faculty of Medicine, Assiut University, Assiut, Egypt; 4 Department of Pathology, College of Medicine, King Saud University, Riyadh, Saudi Arabia; 5 Department of Medical Oncology, South Egypt Cancer Institute, Assiut University, Assiut, Egypt; Fondazione IRCCS Istituto Nazionale dei Tumori, ITALY

## Abstract

**Background:**

The prognostic impact of neoadjuvant chemotherapy (NAC) on the receptor expression status in patients with locally advanced breast cancer (LABC) is still not fully understood. We aimed to evaluate the changes in hormone (estrogen and progesterone) receptor (HR) and human epidermal growth factor receptor 2 (HER2) status post-NAC and their correlation with survival.

**Methods:**

Patients with LABC who have received NAC between 2008 and 2015 and have been followed up till December 2019 at the Oncology Center, King Saud University, KSA were analyzed retrospectively. biomarker analysis of ER, PR & HER2 were done using immunohistochemistry (IHC) and Fluorescent in situ hybridization.

**Results:**

Ninety-one patients fulfilled the inclusion criteria. HR status changed in 21(23.1%) patients, with a significant difference between patients with stable receptors and those with any receptor conversion; p = 0.000. Five (5.5%) initially HER2 negative tumors became HER2 positive and 10 (11%) initially HER2 positive tumors became HER2 negative after NAC. The difference in HER2 expression level before and after NAC was not statistically significant (p = 0.302). Univariate analysis relating patients’ characteristics and 10-years disease-free survival (DFS) showed only significant correlations with the expressions of ER, PR, and any receptor conversion, (ER and/or PR) p< 0.001, p< 0.001, and p = 0.001; respectively. In the univariate analysis, none of the clinicopathological features showed a significant correlation with the OS except for the molecular subtypes P<0.001.

**Conclusions:**

Patients with LABC have significant changes in the ER and PR receptor status following NAC. Post-NAC expressions change of ER and PR (ER and/or PR) are correlated to DFS. Retesting of the hormone receptors should be considered after NAC in Saudi patients with LABC.

## 1. Background

Breast cancer (BC) is the most common malignancy in females in Saudi Arabia, with evidence of a surge in annual incidence from 23.5 /100,000 in 2000 to 34.5 cases/100,000 in 2010 [1, 2]. Neoadjuvant chemotherapy (NAC) followed by definitive surgical resection is the usual therapeutic approach for locally advanced breast cancer (LABC). Besides improving the operability of these patients by downstaging their primary tumors, NAC is a pragmatic approach to preoperatively test chemosensitivity on an individual basis [3, 4].

Performing core needle biopsy (CNB) is a common practice to confirm the diagnosis and determine the presence of immunohistochemical (IHC) markers such as hormone (estrogen and progesterone) receptor (HR) and human epidermal growth factor receptor 2 (HER2), which are important prognostic indicators as well as key factors in the decision-making process regarding further adjuvant therapy [5, 6].

Changes in HR and HER2 receptor status following NAC have been interesting, yet conflicting, research areas for the past couple of years. While several studies suggested that the expression of these receptors is altered after NAC [7–12], others indicated that they remained stable [13, 14]. Moreover, it has been shown that BC patients demonstrating a conversion from HR (+) to HR (−) tended to benefit less from NAC, compared to those with no change or the opposite conversion; from HR (−) to HR (+) [7]. Breast cancer is the most common type of female malignancies in Saudi Arabia with little is known about its pathobiology [15]. Moreover, to the best of our knowledge, there is little information on the prognostic impact of receptor conversion caused post-NAC in patients with LABC. Therefore, we aimed in the current to assess any discordance in the expression profiles of HR and HER2 in patients with residual tumors after NAC, the rate of such discordance (if any), and the prognostic significance of such receptor changes in terms of survival outcomes.

## 2. Materials and methods

### 2.1. Patients, treatment modalities, and samples

Approval of this study was obtained from King Saud University Review Board, King Saud University, KSA. King Saud University Review Board consists of a committee of experts that reviewed and approved the study. The primary endpoint of our study was to evaluate the expression changes of ER, PR, and Her2 neu post neoadjuvant chemotherapy and the secondary endpoint was to correlate these changes with the disease-free survival and overall survival. We have retrospectively collected the clinicopathological data of 120 breast cancer patients and ER, PR, and Her2 neu expression in the pre & post-NAC between January 2008 and December 2015 and have been followed up to December 2019. All patients were at stage II to III with a follow-up duration of more than 6 months.

Only patients who had initial pathologic testing at our hospital and had residual tumor was included. Exclusion criteria included patients with insufficient tumor tissue in post-surgical material either due to complete pathological response or patients who lost regular follow up, patients who had received any type of treatment before NAC, proven metastatic disease before surgery, cases with bilateral breast cancer, male breast cancer, and inflammatory breast cancer were excluded. Patients received one of the following three chemotherapy regimens: The first regimen included 4 cycles of cyclophosphamide and doxorubicin every 15 days, followed by 4 cycles of paclitaxel every 15 days (AC-T), The second regimen included 3 cycles of 5- fluorouracil, epirubicin, and cyclophosphamide, followed by 3 cycles of Taxotere (FEC-D) and the third regimen included 6 cycles of Taxotere, adriamycin, and cyclophosphamide (TAC). After NAC, patients underwent the appropriate surgery approximately 3 weeks after the last chemotherapy cycle and adjuvant radiation therapy. Patients who had a positive IHC for ER and/or PR at any time-point were treated with hormonal therapy regardless of any change in their HR receptor status. Trastuzumab was given to HER2-positive patients for a total of one year.

The residual tumor cells at the primary tumor site (breast) of surgical specimens have been evaluated and the hematoxylin and eosin (H&E) slides of specimens have been reconfirmed by the pathologists. The pathologic complete response (pCR) was achieved in 29 out of 120 patients. Therefore, 91 formalin-fixed paraffin-embedded (FFPE) pre-NAC and matched surgical specimens were included in this study. All patients were followed up every three months for the first year, every 6 months for the next 2 years, and then yearly. Follow-up was completed on December 31, 2019.

### 2.2. Immunohistochemistry

Pre-NAC and surgical FFPE tissue block by immunohistochemistry (IHC) were used to evaluate biomarker status. IHC staining for ER (Confirm anti-ER, rabbit monoclonal primary antibody; clone SP1, Ventana Medical Systems), PR (Confirm anti-PR, rabbit monoclonal primary antibody; clone 1E2; Ventana, and human epidermal growth factor receptor 2 (HER2) (anti-HER2/neu, rabbit monoclonal primary antibody; clone 4B5; Ventana) was performed and according to the manufacturer’s recommendations of the automated Benchmark XT platform (Ventana Medical Systems).

The reporting of ER, PR, and HER2 staining was performed as per the American Society of Clinical Oncology (ASCO) and the College of American Pathologists (CAP) guidelines [16, 17]. In the case of ER and PR, the percentage and intensity of nuclear staining with ER and PR were estimated, and nuclear staining of > 1% of the invasive tumor cells was interpreted as positive. For the study of HER2 status, the protein expression on the membrane of invasive tumor cells was assessed. HER2 staining was graded as per /CAP recommendation from 0 to 3+. Grade 0 and 1+ were considered negative, grade 3+ was considered positive and grade 2+ was considered equivocal. All cases with grade 2+ scores were further evaluated with the fluorescence in situ hybridization (FISH) method. The expression patterns of ER, PR, and HER 2 in the pre-NAC needle biopsies were then compared with the expression patterns of the post-operative specimens.

### 2.3. Statistical analysis

Statistical Package for Social Science (SPSS), version 22 was used for analysis. (PSS Inc., Chicago, IL, USA) Statistical significance was set at < 0.05. Patients with residual disease, at primary tumor site after NAC, was considered as either receptor stable group (having no conversion in either ER, PR, or HER2 status) or any receptor conversion group (having conversion in either PR, ER, or HER2 status). Hormonal receptor & HER2 expression conversion was considered if the receptor status changed from positive to negative or from negative to positive after NAC. The associations between ordinal variables were assessed using Pearson Chi-Square, McNemar test, or Fisher Exact Test in 2x2 tables. Patient prognosis was evaluated through the overall survival (OS). OS is defined as the date from the diagnosis to the date of death from any cause, death from breast cancer, and last follow-up. Disease-free survival (DFS) is defined as the time from diagnosis to distant recurrence, locoregional recurrence, contralateral cancer, secondary cancer, death from cancer. [7]. The Kaplan–Meier method was used to estimate survival. The survival differences were analyzed by the log-rank test. Cox regression analysis was performed to test the effect of major prognostic factors on disease-free survival (DFS) and overall survival (OS).

## 3. Results

### 3.1. Patient characteristics

The clinical characteristics of 91 patients with FFPE of pre-NAC and matched residual disease at the surgical specimens are detailed in Table 1. The median age of our cohort was 47 years (range of 23 to 74 years). The initial (pre-NAC) expression of ER, PR, and HER2 receptors was negative in 22 (24%), 28 (31%), and 59 (65%) of patients, respectively. There was a statistically significant difference in the patient’s clinical characteristics (age, menopausal status), tumor characteristics (stage, histopathology, and grade), pre-NAC expressions of ER, PR, and HER2 receptors, as well as the NAC regimens.

**Table 1:** Clinical characteristics of the cohort (n = 91)*.

Characteristic	Number	%	*P*-value
**Age, years**			
**Median (range)**	47.0 (23.0–74.0)		
**Age groups**			<0.001^¶^
20–40 years	24	26.4	
40–60 years	51	56	
≥ 60 years	16	17.6	
**Menopausal status**			
Premenopausal	57	63	0.021
Postmenopausal	34	37	
**Stage (pre-NAC)**			
II	31	34	0.003
III	60	66	
**Histopathology**			
Invasive ductal	86	95	<0.001^¶^
Invasive lobular	5	5	
**Tumor grade**			
Low	1	1	<0.001^¶^
Intermediate	43	47	
High	47	52	
**Molecular subtypes**			<0.001^¶^
Luminal	47	51.6	
Triple-negative	12	13.2	
ER+PR+HER2+	22	24.2	
ER-PR-HER2+	10	11.0	
**Initial ER status**			
Negative	22	24	<0.001^¶^
Positive	69	76	
**Initial PR status**			
Negative	28	31	<0.001^¶^
Positive	63	69	
**Initial HER2 status**			
Negative	59	65	0.006
Positive	32	35	
**LVI**			
Negative	42	46	0.529
Positive	49	54	
**NAC regimens**			
FEC-D	53	58	<0.001^¶^
AC-T	23	25	
TAC	15	17	

NAC; neoadjuvant chemotherapy, ER; estrogen receptors, PR; progesterone receptors, HER2; Human epidermal growth factor receptor-2, LVI; lymphovascular invasion, FEC-D; 5- fluorouracil, epirubicin, cyclophosphamide-Taxotere, AC-T; cyclophosphamide, doxorubicin-paclitaxel, TAC; Taxotere, Adriamycin-cyclophosphamide

* Non-parametric One-sample Chi-square test

^¶^ Non-parametric Binominal test

### 3.2. Conversion in receptor status

The statuses of hormone receptors in the 91-matched initial pre-NAC biopsies and post-NAC surgical specimens were analyzed using the McNemar test. The expression of ER remained the same in 74 (81.3%) specimens. One initially ER-negative tumor turned ER-positive and 16 (17.5%) initially ER-positive ones turned into ER-negative post NAC. The difference in ER expression pre and post-NAC was highly significant (p<0.001). PR expression remained the same in 76 (83.5%) of the specimens. Three (3.3%) initially PR negative tumors turned PR positive and 12 (13.2%) initially PR positive tumors turned PR negative post-NAC. The difference in PR expression pre and post-NAC was statistically significant (p = 0.035). In sum, the expression of both ER and PR remained the same in 70 (77%) specimens, while there was a conversion of both ER and PR in 21 (23%) ones. Four (4.4%) specimens were ER stable PR converted, while 6 (6.6%) were PR stable ER converted. Thus, 21 (23.1%) of 91 patients had ER and/or PR conversion after NAC; with a highly significant difference between patients with stable receptors and those with any receptor (ER and/or PR) conversion; p< 0.001. For HER2 expression, it remained the same in 76 (83.5%) specimens. Five (5.5%) initially HER2 negative tumors became HER2 positive and 10 (11%) initially HER2 positive tumors became HER2 negative after NAC. The difference in HER2 expression level before and after NAC was not statistically significant (p = 0.302). Table 2 shows the details of receptor status conversion among the study cohorts.

**Table 2:** Baseline and Post-NAC hormonal receptors’ status among the study cohorts (n = 91).

**Changes in estrogen receptor (ER) status post-NAC**			
**Pre-NAC/Post-NAC**	**No**	**%**	***P*-value**
ER-/ER-	21	23.0	p<0.001^¶^
ER-/ER+	1	1.0	
ER+/ER-	16	17.5	
ER+/ER+	53	48.5	
**Changes in progesterone receptor (PR) status post-NAC**			
**Pre-NAC/Post-NAC**	**No**	**%**	***P*-value**
PR-/PR-	25	27.4	0.035^¶^
PR-/PR+	3	3.3	
PR+/PR-	12	13.2	
PR+/PR+	51	56.1	
**Any receptor changes post-NAC**			
**Receptors stable vs conversion**	**No**	**%**	***P*-value**
Both ER&PR stable	70	77.0	P<0.001^¶^
Both ER&PR converted	11	12.0	
ER stable PR converted	4	4.4	
PR stable ER converted	6	6.6	
**Changes in HER2 receptor (HER2) status post-NAC**			
**Pre-NAC/Post-NAC**	**No**	**%**	***P*-value**
HER2-/HER2-	54	59.3	0.302^¶^
HER2-/HER2+	5	5.5	
HER2+/HER2-	10	11.0	
HER2+/HER2+	22	24.2	

^¶^McNemar test

### 3.3. Survival analysis and prognostic impact of HR conversion

**Fig 1:**
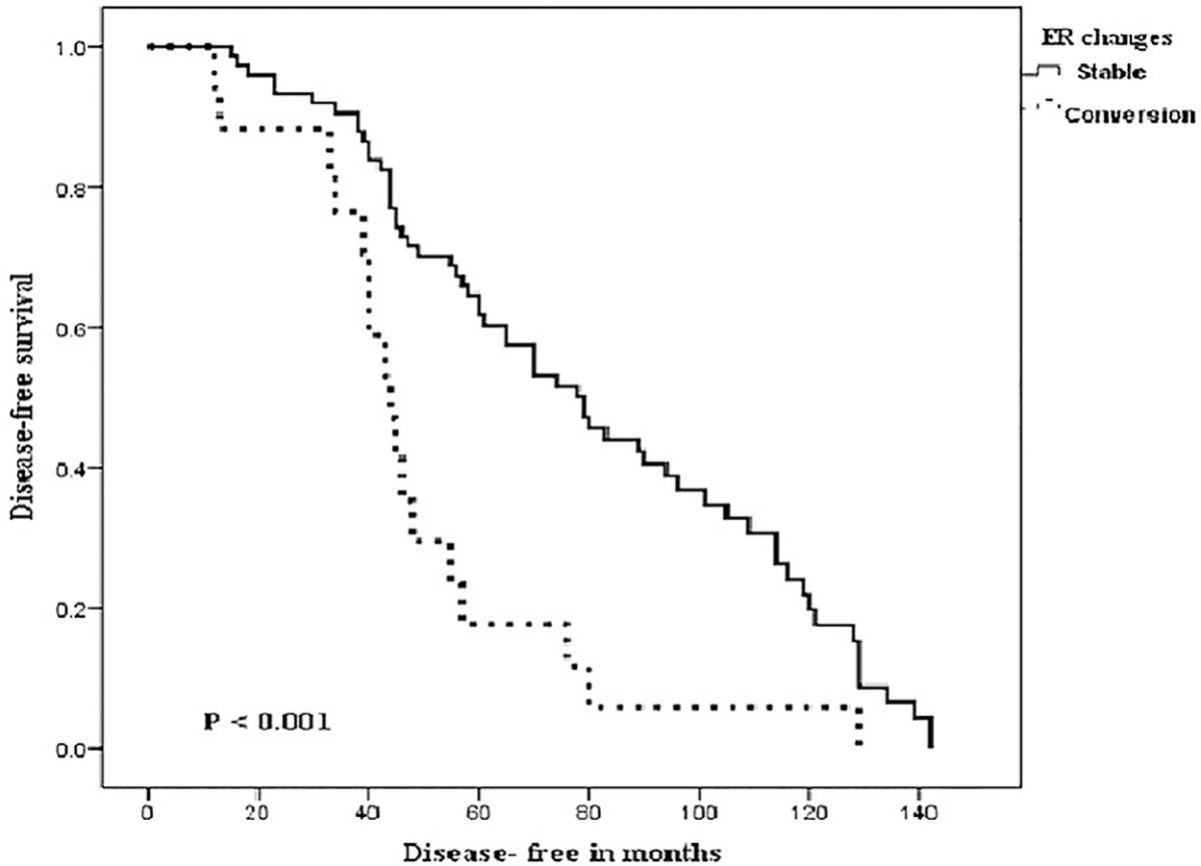
Kaplan-Meier curves of Disease-Free Survival (DFS) in patients with stable and converted estrogen receptors (ER) following neoadjuvant chemotherapy. Log-rank test was significant (P < 0.001).

**Fig 2:**
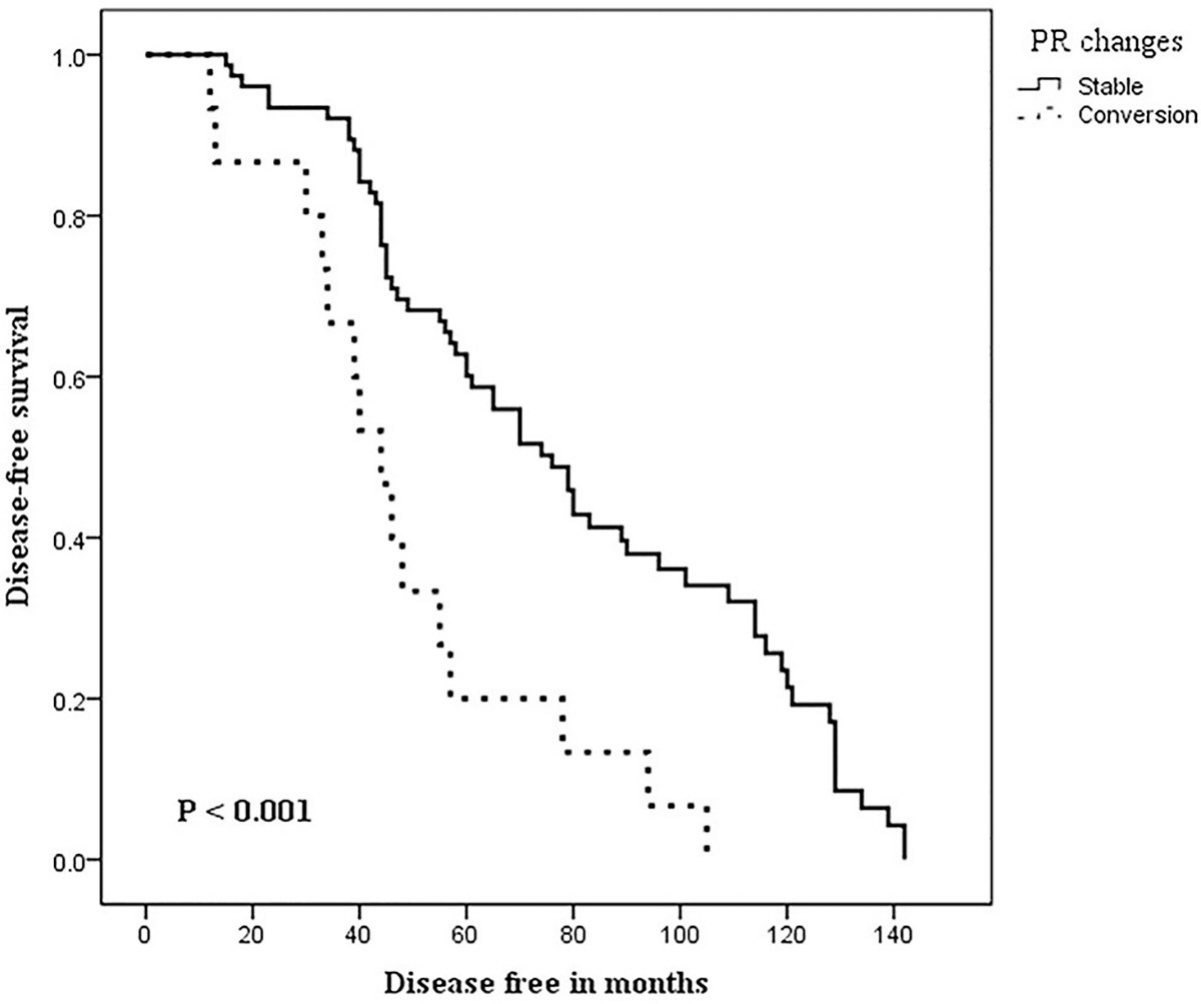
Kaplan-Meier curves of Disease-Free Survival (DFS) in patients with stable and converted Progesterone Receptors (PR) following neoadjuvant chemotherapy. Log-rank test was significant (P < 0.001).

**Fig 3:**
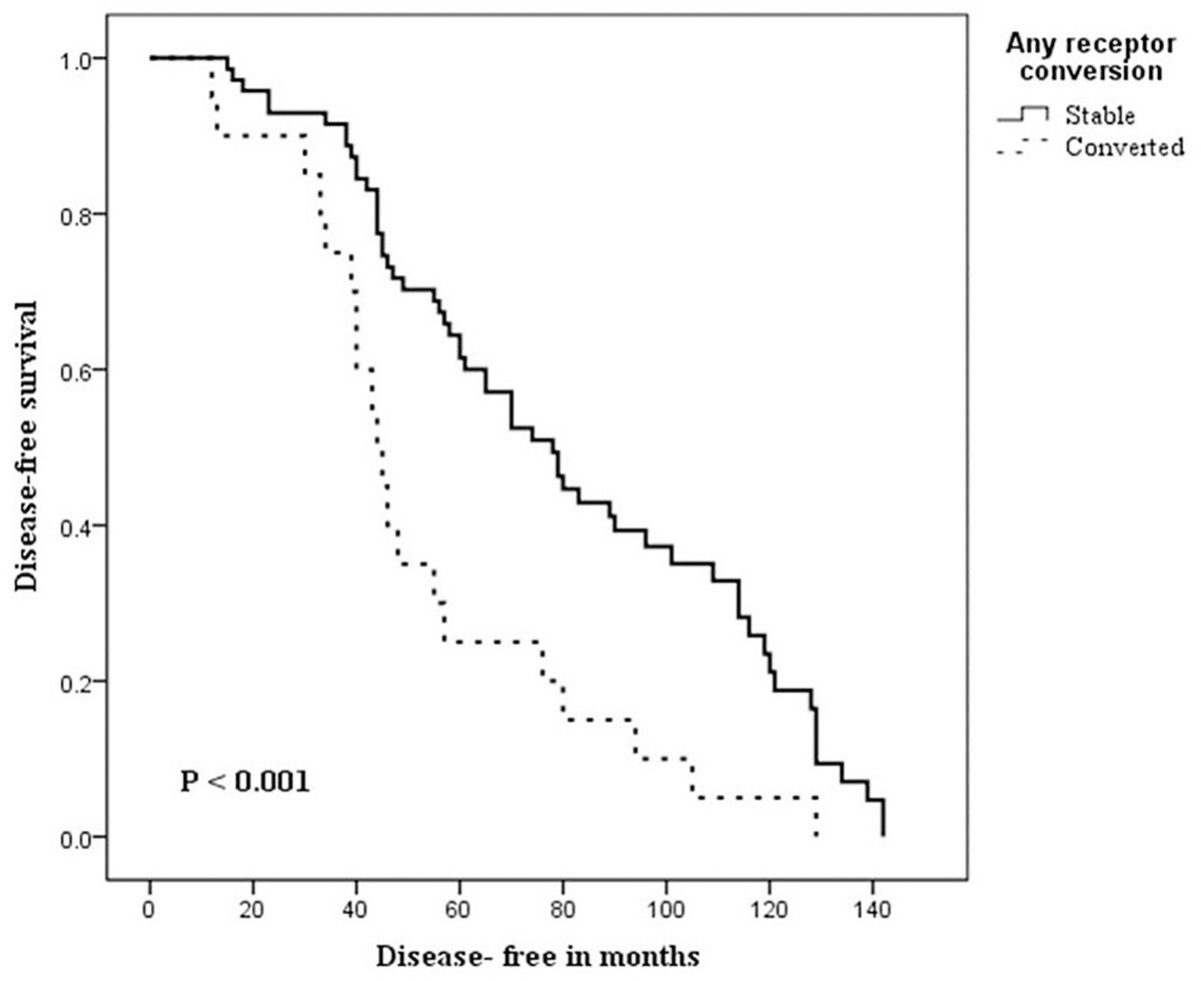
Kaplan-Meier curves of Disease-Free Survival (DFS) in patients with stable and converted any receptor (ER and/or PR) statuses following neoadjuvant chemotherapy. Log-rank test was significant (P< 0.001).

During the follow-up time (range, 16–149 months), 23 (25.3%) of 91 patients had died, and 13 (14.3%) of 91 patients had experienced disease recurrence. The median overall survival was 77.8 months, while the median DFS was 67.4 months. The relationship between the patients clinicopathological and post-NAC receptor changes on one hand and both 10-years DFS and OS, on the other hand, is shown in Tables 3 and 4. In Table 3, univariate analysis between the patient characteristics and DFS showed significant relations with the post-NAC expressions of ER, PR, and any receptor conversion and molecular subtypes, log-rank test; 12.338, p <0.001, 14.296, p <0.001, 10.547, p <0.001 and 19.934, p <0.001; respectively. However, in multivariate Cox regression analysis, none of these variables were independently related to 10-years DFS. Kaplan-Meier curves for DFS in the patients’ groups of stable and converted ER, PR, and any receptor are shown in Figs 1–3, respectively. The differences among the curves were statistically significant as determined by the log-rank test (P <0.001, P <0.001, and p<0.001), respectively. In Table 4, univariate analysis between the patient characteristics and the 10-years OS showed that only molecular subtypes had a significant relationship with overall survival, log-rank test 21.197, p = <0.001.

**Table 3:** The effects of clinicopathological features on 10-years Disease-Free Survival (DFS); univariate and multivariate analyses.

**Characteristic**	**N (%)**	**10-years Disease-free survival (DFS)**			
		**Univariate**		**Multivariate**	
		**HR (95%) CI)**	***P***	**HR (95% CI)**	***P***
**Age groups**					
20–40 y	24 (26)	0.278 (0.396–2.934)	0.840		
40–60 y	51 (56)				
≥ 60 y	16 (18)				
**Menopausal status**					
Premenopausal	57 (63)	1 1	0.143		
Postmenopausal	34 (37)	. 0.734 (0.263–2.053)			
**Stage (before NAC)**					
II	31 (34)	0 0.664 (0.291–2.453)	0.293		
III	60 (66)				
**Histopathology**					
Invasive ductal	86 (95)	1 0.452 (0.482–1.882)	0.787		
Invasive lobular	5 (5)				
**Tumor grade**					
Low	1 (1)	1 0.631 (0.682–2.221)	0.652		
Intermediate	43 (47)				
High	47 (52)				
**Molecular subtypes**					
Luminal	47 (52)	2.775 (1.104–4.900)	<0.001	1.835 (0.997–3.532)	0.051
Triple negative	12(13)				
ER+PR+HER2+	22 (24)				
ER-PR-HER2+	10 (11)				
**Post-NAC ER status**					
Stable	74 (82)	1 1	<0.001	1	0.196
Converted	17 (18)	3.088 (1.815–5.253)		1.726 (0.632–4.363)	
**Post-NAC PR status**					
Stable	76 (84)	1 1	<0.001	1	0.053
Converted	15 (16)	1 3.855 (1.275–11.661)		2.410 (0.997–5.827)	
**Any receptor change**					
Stable	70 (77)	1	<0.001	1	0.618
Converted	21 (23)	2.189 (0.052–1.690)		0.684 (0.154–3.044)	
**Post-NAC HER2 status**					
Stable	76 (84)	1 1	0.143		
Converted	15 (16)	0.974 (0.468–2.030)			
**LVI**					
Negative	42 (46)	1 1	0.441		
Positive	49 (54)	0.723 (0.443–1.527)			
**NAC regimens**					
FEC-D	53 (58)	1 0.620 (0.582–1.981)	0.629		
AC-T	23 (25)				
TAC	15 (17)				

**Table 4:** The effects of clinicopathological features on Overall Survival (OS); univariate analysis.

Characteristic	N (%)	10-years Overall survival (OS)	
		Univariate analysis	
		HR (95% CI)	*P*
**Age groups**	24 (26)	0.878 (0.396–2.934)	0.433
20–40 y	51 (56)		
40–60 y	16 (18)		
≥ 60 y	24 (26)		
**Menopausal status**			
Premenopausal	57 (63)	1	0.551
Postmenopausal	34 (37)	0.640 (0.515–1.714)	
**Stage (before NAC)**			
II	31 (34)	1.578 (0.948–5.565)	0.092
III	60 (66)		
**Histopathology**			
Invasive ductal	86 (95)	0.927 (0.296–2.234)	0.161
Invasive lobular	5 (5)		
**Tumour grade**			
Low	1 (1)	0.667 (0.496–1.924)	0.272
Intermediate	43 (47)		
High	47 (52)		
**Molecular subtypes**		2.678 (0.948–7.565)	<0.001
Luminal	47 (52)		
Triple negative	12(13)		
ER+PR+HER2+	22 (24)		
ER-PR-HER2+	10(11)		
**Post-NAC ER status**			
Stable	74 (82)	1	0.062
Converted	17 (18)	2.075 (1.104–3.900)	
**Post-NAC PR status**			
Stable	76 (84)	1	0.065
Converted	15 (16)	1.620 (0.423–4.636)	
**Any receptor change**			
Stable	70 (77)	1	0.053
Converted	21 (23)	1.987 (0.083–1.810)	
**Post-NAC HER2 status**			
Stable	76 (84)	1	0.070
Converted	15 (16)	1.630 (0.631–2.805)	
**LVI**			
Negative	42 (46)	1	0.413
Positive	49 (54)	0.887 (0.396–2.544)	
**NAC regimens**			
FEC-D	53 (58)	0.351(0.353–1.197)	0.925
AC-T	23 (25)		
TAC	15 (17)		

## 4. Discussion

To the best of the authors’ knowledge, this is the first study that addresses the conversion in the hormonal status (pre-and post-NAC) and its prognostic significance in a well-characterized cohort of Saudi patients with LABC. Our results showed a conversion rate of 23.1% with a highly significant difference between patients with stable receptors and those with any receptor conversion. There was a prognostic influence of these hormonal changes on the 10-years DFS but none of them were independently related. There is a significant surge in the incidence of BC among our cohort, which occurs at an earlier age than in western countries [2].

Neoadjuvant chemotherapy (NAC) or primary systemic therapy is considered the standard treatment for LABC aiming towards a reduction in tumor size, increasing the chances of conservative surgery, and improvement of the cosmetic outcome. [3]. Besides, beyond initiating an early systemic treatment for clinically undetectable micro-metastases, NAC also provides an opportunity to evaluate the tumor sensitivity to different chemotherapeutic regimens [4].

It has been reported that discordance of hormonal expression before and after NAC ranges from 2.5 to 51.7% and the conversion of positive Her 2 neu expression to negative one was reported in 43% of patients with BC [18–22]. Several molecular mechanisms have been proposed to mediate primary or acquired resistance to ETs in HR+ BC. These mechanisms include mutations in the ESR1 gene, which encodes ERα, or alterations in the genes of the mitogen-activated protein kinase (MAPK) pathway (e.g., activating ERBB2 mutations and NF1 loss-of-function mutations) or ESR1 transcriptional regulators [23, 24].

In the current study, we had observed a conversion rate of 23.1% which is in concordance with those previously reported figures, particularly those of Tacca *et al* [18] and Yang *et al* [19]. Several studies have suggested that the expression of these receptors is altered after NAC [7–12], others indicated that they remained stable [13, 14]. Two studies [9, 25] have summarized data and concluded that NAC seemed able to change ER and PR receptors expression and status, but HER2 amplification appeared to be more stable. Discordance of the HR status was reported in 8–33% of the patients, and the authors recommended that retesting the receptor status of the residual tumor after NAC should be considered to improve the future tailored adjuvant therapies [8]. In a pooled meta-analysis of 14 eligible studies, Zhang *et al* [9] had demonstrated that HR status (ER and PR) can be significantly changed with the use of NAC, with significant differences in the reported ER and PR status; however, the effect of NAC on HER2 and Ki67 were not statistically significant. In the current retrospective analysis of 91 patients, our results confirmed the previously reported results [7–12], we observed conversion of HR expression status after NAC in 23.1% of our cohorts. More importantly, these hormonal changes had a prognostic significance as they were correlated significantly with the 10-years DFS. Interestingly, 17.5% and 13.2% of ER-positive and PR-positive tumors changed to ER-negative and PR-negative, while only 1% and 3.3% of ER-negative and PR-negative tumors changed to ER-positive and PR-positive respectively. Moreover, these results are similar to what was reported in the previous studies [10, 20]. Except for random changes due to small-sized samples, laboratory procedures, observer variability, the possible mechanisms for a change in receptor status or expression in BC cells after chemotherapy are complicated [20]. Intra-tumoral heterogeneity might lead to several different clones with varied phenotypes within individual tumors [22]. Even within the same tumor, some clones are HR (+) while others are HR (−). Likewise, HER2 (+) cells are not evenly distributed within individual tumors. The sensitivity to chemotherapy also differs between different clones, with HR (−) tumor cells being more sensitive to chemotherapy than HR (+) tumors cells, which are aptly named insensitive tumor cells [26], and are left behind as part of the residual disease after NAC [8, 18]. Another established mechanism of HR status conversion is the ER downregulation caused by NAC itself. It is reported that chemotherapy can suppress ovarian function and adrenal glands [27, 28], and the decrease in the circulating levels of hormone resulting from this suppression might alter the HR status of residual tumors from (+) to (−) post NAC [8]. This mechanism is considered to be the main reason for the switch of HR (+) to HR (−) post NAC, which was observed frequently in our cohorts. Other explanations for the conversion of receptor status include genetic mutations [29], staining techniques, and statistical errors [11].

The 10-years survival analysis of our cohort revealed worse DFS in patients with converted receptor status (ER, PR, and any receptor change) than those with stable statuses, respectively. Moreover, the DFS was significantly correlated with the molecular subtypes but in Cox Regression analysis none of these factors were independently correlated.

Except for molecular subtypes, no significant correlation was found for the OS. There is a dearth of literature on the prognostic value of changed receptor status. Few studies have demonstrated the relations between HR conversion and treatment response, but discordant conclusions were observed. Chen *et al* [7] reported that patients with an HR (+) to (-) switch, benefit less from endocrine therapy compared to those whose HR status remains stable. In contrast, Tacca *et al* [18] and Buchholz *et al* [30] observed that there were no significant differences in PFS and OS rates between endocrine therapy-administered to patients with HR-negative switch lesions, and those with HR-positive lesions, both pre and post-NAC. Regardless, the authors demonstrated that a positive switch of the HR-status could be an indicator for a better outcome. Of note, despite the differences in the patients’ numbers and epidemiologic characteristics, our finding of prognostic significance between HR conversion and only DFS are similar to those reported by the recent large retrospective trial came from MD Anderson Cancer Center [31] which reported that is a total of 398 women-any receptor change was correlated with increased relapse-free survival, but no correlation with OS. Still, however, our findings are different from those observed by Chen *et al* [7] and Ozmen *et al* [32] who reported that change in HR status was an independent predictor for a poorer DFS as well as OS.

The strengths of our study are being in agreement with some published data, significant changes in IHC expression of ER, PR in LABC post neoadjuvant chemotherapy has been detected. More importantly, post-NAC expressions of ER and PR were independently related to DFS. Inevitably, the current study has some limitations of being a retrospective study, including a relatively small number of patients and the existence of tumor heterogeneity has led to concerns that core biopsies may not be representing the whole tumor tissue as they are often restricted to the superficial aspects of the tumor.

## 5. Conclusion

Patients with LABC had significant changes in the ER and PR receptor expression status after neoadjuvant chemotherapy. Post-NAC expressions of ER and PR were correlated with DFS. Until more comparable and/or prospective studies are carried out, retesting of the hormone receptors should be considered after NAC in patients with LABC. Further prospective studies enrolling a larger number of patients are warranted to understand the relationship between NAC and hormonal pathways and to explore the strategies of more therapeutic benefit.

## Supporting information

S1 File (XLSX)
